# Schrödinger's Cat Paradox: *Bartonella* Serology Cannot Be Used to Speciate *Bartonella* Endocarditis

**DOI:** 10.1093/ofid/ofad436

**Published:** 2023-08-16

**Authors:** Carl Boodman, Nitin Gupta

**Affiliations:** Department of Internal Medicine, Division of Infectious Diseases, University of Manitoba, Winnipeg, Manitoba, Canada; Department of Clinical Sciences, Unit of HIV and Neglected Tropical Diseases, Institute of Tropical Medicine, Antwerp, Belgium; Department of Clinical Sciences, Unit of HIV and Neglected Tropical Diseases, Institute of Tropical Medicine, Antwerp, Belgium; Department of Infectious Disease, Kasturba Medical College, Manipal Academy of Higher Education, Manipal, India

To the Editor—We read with great interest Ordaya and colleagues' recent article titled “Let the Cat Out of the Heart,” describing the clinical characteristics of 16 cases of *Bartonella* endocarditis [1]. We congratulate the authors on an intriguing case series that highlights *Bartonella* endocarditis's association with renal failure, embolization, and PR3-ANCA positivity. Publishing case-level data on *Bartonella* endocarditis is increasingly relevant in light of the 2023 updates to the modified Duke criteria that include *Bartonella* diagnostics as a major endocarditis criterion [2].

However, we have concerns that the article, including the witty title, may mislead readers to consider *Bartonella henselae* as the predominant etiology of *Bartonella* endocarditis without providing species-level evidence to support this claim. The 16 cases described were diagnosed by *Bartonella* serologic positivity, with 7 patients confirmed to *Bartonella* genus level by *Bartonella* polymerase chain reaction (PCR) on explanted cardiac tissue. Species-specific PCR targets such as 16S rRNA, *ribC*, *rpoB*, and *gltA* genes were not performed [3]. While the authors mention *Bartonella* species other than *B henselae* in the introduction, they proceed to focus on *B henselae*, emphasizing cat exposure and *Ctenocephalides* flea vectors. “Cat exposure” was reported in 62.5% of the described cases, but the authors failed to define what this exposure meant. Were all “cat exposures” scratches or simply a recollection of a feline in the vicinity? The authors also failed to include important risk factors for *Bartonella quintana*, such as homelessness, pediculosis, or immigration from a low-income country [4, 5]. This may reflect heuristic, confirmation, and premature closure biases [6]; the authors imply that any “cat exposure” is a mental shortcut for *B henselae* infection without sufficient information to exclude alternative *Bartonella* species.

The authors appropriately recognize the limitations of serology, recognizing that *Bartonella* endocarditis may cause reactive *Coxiella burnetii* serology. Eight of the 16 patients in this study resulted in false-positive *C burnetii* serology. Analogously, *Bartonella* serology lacks specificity and cross-reacts between *Bartonella* species. *Bartonella quintana* and *B henselae* commonly cause serologic positivity for the other species, with similar titers [7]. In Manitoba, a Canadian province that borders Minnesota (jurisdiction of the authors), *B quintana* was a more common cause of *Bartonella* serologic positivity than *B henselae* [7]. One cannot assume *Bartonella* species based on serology.

In the introduction, Ordaya et al appropriately describe homelessness, human immunodeficiency virus (HIV), and alcohol use as risk factors for *B quintana* infection. However, only HIV and alcohol use were reported in their Table 1; current homelessness, previous homelessness, and shelter exposure were not described. Moreover, it is increasingly recognized that *B quintana* is endemic to many low-income countries [5, 8]. Thus, immigration history is an important and often neglected risk factor for *B quintana* infection that was not described here. Last, *B quintana* has previously been isolated from cats, and human cases of *B quintana* have occurred after a feline bite [9, 10]. Cat exposure alone is not sufficient to confirm *B henselae* among endocarditis cases with *Bartonella* serologic positivity.

In quantum mechanics, Schrödinger's cat is a thought experiment to demonstrate quantum superposition [11]. In this parable, a cat, a flask of poison, and a radioactive source are sealed in a box. If radioactivity is detected, the poison is released from the flask, eventually killing the cat. After a period of time, until one looks into the box, the cat can be both dead and alive. Only after opening the box can one identify either an alive or dead cat. Using this analogy, if we do not open the box and use molecular testing to identify the *Bartonella* species, *Bartonella* endocarditis may be caused by either *B henselae* or *B quintana* (or another species) and may or may not be linked to a cat.

## Note

***Potential conflicts of interest.*** All authors: No reported conflicts.

## References

[1] Ordaya EE , Abu Saleh OM, Mahmood M, et al “Let the cat out of the heart”: clinical characteristics of patients presenting with blood culture–negative endocarditis due to *Bartonella* species. Open Forum Infect Dis 2023; 10:ofad293. 10.1093/ofid/ofad293 PMC10372712 37520412

[2] Fowler VG Jr , Durack DT, Selton-Suty C, et al The 2023 Duke-ISCVID criteria for infective endocarditis: updating the modified Duke criteria. Clin Infect Dis 2023; 77:518–26.37138445

[3] McCormick D , Rassoulian-Barrett S, Hoogestraat D, et al *Bartonella* spp. infections identified by molecular methods, United States. Emerg Infect Dis J 2023; 29:467–76. 10.3201/eid2903.221223 PMC9973681 36823096

[4] Foucault C , Brouqui P, Raoult D. *Bartonella quintana* characteristics and clinical management. Emerg Infect Dis J 2006; 12:217–23. 10.3201/eid1202.050874 PMC3373112 16494745

[5] Tasher D , Raucher-Sternfeld A, Tamir A, Giladi M, Somekh E. *Bartonella quintana*, an unrecognized cause of infective endocarditis in children in Ethiopia. Emerg Infect Dis 2017; 23:1246–52. 28730981 10.3201/eid2308.161037 PMC5547792

[6] Vick A , Estrada CA, Rodriguez JM. Clinical reasoning for the infectious disease specialist: a primer to recognize cognitive biases. Clin Infect Dis 2013; 57:573–8. 23595833 10.1093/cid/cit248

[7] Boodman C , Wuerz T, Lagacé-Wiens P, et al Serologic testing for *Bartonella* in Manitoba, Canada, 2010–2020: a retrospective case series. CMAJ Open 2022; 10:E476–82. 10.9778/cmajo.20210180 PMC9177198 35640989

[8] Pecoraro AJ , Herbst PG, Doubell AF. Infective endocarditis in Africa: an urgent call for more data. Lancet Glob Health 2022; 10:e8–9. 34919860 10.1016/S2214-109X(21)00489-7

[9] La VD , Tran-Hung L, Aboudharam G, Raoult D, Drancourt M. *Bartonella quintana* in domestic cat. Emerg Infect Dis 2005; 11:1287–9. 16102321 10.3201/eid1108.050101 PMC3320506

[10] Breitschwerdt EB , Maggi RG, Sigmon B, Nicholson WL. Isolation of *Bartonella quintana* from a woman and a cat following putative bite transmission. J Clin Microbiol 2007; 45:270–2. 17093037 10.1128/JCM.01451-06 PMC1828989

[11] Monroe C , Meekhof DM, King BE, Wineland DJ. A “Schrödinger cat” superposition state of an atom. Science 1996; 272:1131–6. 8662445 10.1126/science.272.5265.1131

